# Association of liver function and prognosis in patients with severe fever with thrombocytopenia syndrome

**DOI:** 10.1371/journal.pntd.0012068

**Published:** 2024-04-16

**Authors:** Shaoqiu Zhang, Jian Wang, Qun Zhang, Yifan Pan, Zhiyi Zhang, Yu Geng, Bei Jia, Yuanyuan Li, Yali Xiong, Xiaomin Yan, Jie Li, Huali Wang, Chao Wu, Rui Huang

**Affiliations:** 1 Department of Infectious Diseases, Nanjing Drum Tower Hospital, Affiliated Hospital of Medical School, Nanjing University, Nanjing, Jiangsu, China; 2 Institute of Viruses and Infectious Diseases, Nanjing University, Nanjing, Jiangsu, China; 3 Department of Infectious Diseases, Affiliated Zhongda Hospital of Southeast University, Nanjing, Jiangsu, China; 4 Department of Infectious Diseases, Nanjing Drum Tower Hospital Clinical College of Nanjing Medical University, Nanjing, Jiangsu, China; 5 Department of Infectious Diseases, Nanjing Drum Tower Hospital Clinical College of Nanjing University of Chinese Medicine, Nanjing, Jiangsu, China; 6 Department of General Practice, Nanjing Second Hospital, Nanjing University of Chinese Medicine, Nanjing, Jiangsu, China; Uniformed Services University: Uniformed Services University of the Health Sciences, UNITED STATES

## Abstract

**Objectives:**

Severe fever with thrombocytopenia syndrome (SFTS) is an epidemic emerging infectious disease with high mortality rate. We investigated the association between liver injury and clinical outcomes in patients with SFTS.

**Methods:**

A total of 291 hospitalized SFTS patients were retrospectively included. Cox proportional hazards model was adopted to identify risk factors of fatal outcome and Kaplan-Meier curves were used to estimate cumulative risks.

**Results:**

60.1% of patients had liver injury at admission, and the median alanine transaminase, aspartate aminotransferase (AST), alkaline phosphatase (ALP), and total bilirubin (TBil) levels were 76.4 U/L, 152.3 U/L, 69.8 U/L and 9.9 μmol/L, respectively. Compared to survivors, non-survivors had higher levels of AST (253.0 U/L vs. 131.1 U/L, P < 0.001) and ALP (86.2 U/L vs. 67.9 U/L, P = 0.006), higher proportion of elevated ALP (20.0% vs. 4.4%, P < 0.001) and liver injury (78.5% vs. 54.9%, P = 0.001) at admission. The presence of liver injury (HR 2.049, P = 0.033) at admission was an independent risk factor of fatal outcome.

**Conclusions:**

Liver injury was a common complication and was strongly associated with poor prognosis in SFTS patients. Liver function indicators should be closely monitored for SFTS patients.

## Author summary

Severe fever with thrombocytopenia syndrome (SFTS) is an emerging epidemic infectious disease with high mortality rate. However, the prevalence of liver injury and its association with the prognosis of SFTS patients has not been well characterized. In this multi-center cohort study, we found that the incidence of liver injury in non-survivors was significantly higher than that in survivors both at admission and during hospitalization. Patients with liver injury at admission had higher cumulative mortality risk than those without liver injury. Our study demonstrated that the presence of liver injury is strongly associated with poor prognosis in SFTS patients. Liver function should be closely monitored in SFTS patients, which may contribute to better management of SFTS.

## Introduction

Severe fever with thrombocytopenia syndrome (SFTS) is an emerging epidemic infectious disease caused by a novel bunyavirus (SFTS virus, SFTSV) which is later reclassified as Dabie bandavirus. It first emerged in China in 2010 with an estimated mortality rate up to 30% [1,2] and had been reported in Korea [3], Japan [4], Thailand [5] and the United States [6]. Patients with SFTS mainly present with non-specific symptoms including fever, thrombocytopenia, leukocytopenia and other systemic symptoms including haemorrhagic presentations [7]. Among critically ill SFTS patients, the clinical symptoms deteriorate quickly within a week after symptoms onset, and most patients died of disseminated intravascular coagulation (DIC) and multiorgan failure including liver injury [8–10].

Liver injury as a complication has been reported in SFTS patients [11,12]. It is reported that a majority of SFTS patients had abnormal liver indicators [13]. Liver injury occurs in the early stage of SFTS [7], and several studies [2,14] have reported that liver function abnormality might be a risk factor of poor outcome. It is also reported that SFTS patients had pathological feature of liver lobular necrosis and mild portal fibrosis and SFTSV antigens was tested positive in the liver tissues [15], suggesting that liver may be a target organ of SFTSV. The liver might play a significant role in the pathogenesis of SFTSV infection.

However, the association of liver function and clinical outcomes in SFTS has not been well established. Therefore, we conducted a multi-center retrospective cohort study to assess the prevalence of liver injury and its association with the prognosis of SFTS patients.

## Materials and methods

### Ethics statement

The protocol has acquired the approval of the human ethics committee of Nanjing Drum Tower Hospital (No. 2022-238-02). The study was performed in accordance with the Declaration of Helsinki. A waiver of informed consent was granted by the ethics committee of Nanjing Drum Tower Hospital due to a retrospective design.

### Patients and study design

In this multi-center, retrospective cohort study, consecutive SFTS patients admitted to three hospitals (Nanjing Drum Tower Hospital, The Second Hospital of Nanjing, and Zhongda Hospital Southeast University) between October 2010 and August 2022 were included. The clinical data were extracted from medical records using a structured data frame, including demographic information, underlying comorbidities, clinical manifestation and course, treatment regimens, and laboratory tests results. The primary outcome was all-cause mortality. The follow-up time of survivor was calculated from the day of admission to discharge, and the duration of non-survivors was calculated from the day of admission to that of death.

### Inclusion and exclusion criteria

Patients with laboratory-confirmed SFTSV infection were included in the study. The diagnosis of SFTSV infection was confirmed by at least one of the criteria as follows: (1) positive results of serum viral RNA, (2) seroconversion or a 4-fold or higher increase of antibody titers between two serum samples collected at an interval of more than two weeks, (3) isolation of SFTSV from cell culture. Those who were positive for other tick-borne pathogens or were not confirmed by laboratory examinations were excluded.

### Definition of chronic liver diseases, liver abnormality and liver injury

Chronic liver diseases (CLD) are characterized by the gradual destruction of liver tissue over time, and includes liver diseases that are caused by chronic inflammation with or without cirrhosis and hepatocellular carcinoma (HCC) [16,17]. The upper limit of normal values (ULNs) of alanine aminotransferase (ALT), aspartate transaminase (AST), alkaline phosphatase (ALP), γ-glutamyl transpeptidase (GGT), and total bilirubin (TBil) were 40 U/L, 40 U/L, 185 U/L, 35 U/L for female and 50 for male, and 28 μmol/L, respectively. Any of the above indicators of liver function tests exceed the ULNs would be considered as liver abnormality. Liver injury was defined as an elevation in ALT or AST of at least 3×ULN, or an elevation in ALP or TBil of at least 2×ULN [14,18–20]. Liver abnormalities were further classified into different patterns based on the results of liver function tests: (1) Hepatocellular type refers to the pattern of ALT or AST levels above ULN and ALP level within the normal range; (2) Cholestatic type refers to the elevation of ALP levels but with normal AST and ALT levels; (3) Mixed type refers to the elevation of both ALP and ALT/AST levels; (4) Others refer to patterns not matched with types of liver abnormalities mentioned above [18–20]. Hazardous alcohol consumption was defined as at least 20 g and 40 g of alcohol per day for women and men, respectively [21].

### Statistical analysis

Continuous variables were described as median (interquartile range, IQR) for the skewed distribution of the data, and categorical variables were expressed as frequencies or proportions. Continuous variables were compared by independent sample T test or Mann Whitney U test, and categorical variables were compared by Chi-square or Fisher exact test between survivors and non-survivors. Cox proportional hazards analysis was used to investigate the associations between liver abnormalities and fatal outcome during hospitalization. Demographic, clinical and laboratory parameters were included in univariate cox analysis. Baseline parameters that showed a univariate relationship with mortality and that consider clinically relevant were further included in multivariate analysis. Confounding factors were evaluated by clinically relevant and descriptive statistics from our cohort through the use of directed acyclic graphs. The Kaplan-Meier curves were adopted to compare cumulative survival rates among patients stratified by the presence of liver injury or chronic liver diseases (CLD) at admission. Two-factor analysis of variance (ANOVA) was used to evaluate the moderating effect for the association of liver function and survival outcome. All statistical analysis were conducted with SPSS version 25.0 (IBM, Armonk, NY, USA) and all graphs were plotted by R version 4.2.1 (R Foundation, Vienna, Austria; www.R-project.Org). A P<0.05 was considered as statistically significant.

## Results

### Baseline characteristics of patients with SFTS

A total of 291 patients with SFTS were included in this study. Male patients represented 51.9% and the median age was 63.0 (52.0, 71.0) years. The median days from symptom onset to hospital admission were comparable between survivors and non-survivors (7.0 days vs. 7.0 days, P = 0.265). Patients were followed for a median of 10 (IQR 7.0, 14.0) days. Sixty-five (22.3%) patients died after a median time of 6.0 (2.0, 9.0) days from admission to death. Two hundred and twenty-six survived patients had a median time of hospitalization for 11.0 (8.0, 14.0) days.

In comparison, non-survivors were older than survivors (69.0 years vs. 60.0 years, P<0.001). Survivors had higher median levels of PLT (52.0 ×10^9^/L vs. 35.0 ×10^9^/L, P<0.001), ALB (33.8 g/L vs. 30.1 g/L, P<0.001), and lower levels of Cr (58.5 μmol/L vs. 80.0 μmol/L, P<0.001), PT (11.2 s vs. 12.2 s, P<0.001) and INR (1.0 vs. 1.1, P<0.001) than non-survivors at admission. The complications of disturbance of consciousness, respiratory failure and shock were more commonly observed in the non-survivors (Table 1). More non-survivors received mechanical ventilation and blood purification treatment. The use of corticosteroid and intravenous immunoglobulin (IVIg) were more frequently observed in non-survivors (Table 1).

**Table 1:** The clinical features of patients with severe fever with thrombocytopenia syndrome between survivors and non-survivors.

	All patients (n = 291)	Survivors (n = 226)	Non-survivors (n = 65)	P value
**Age (yr)**	63.0 (52.0, 71.0)	60.0 (50.8, 69.0)	69.0 (57.5, 72.0)	<0.001
**Male (%)**	151 (51.9)	121 (53.5)	30 (46.2)	0.294
**Days from symptom onset to hospitalization**	7.0 (5.0, 9.0)	7.0 (5.0, 9.0)	7.0 (6.0, 10.0)	0.265
**Days of hospitalization**	10.0 (7.0, 14.0)	11.0 (8.0, 14.0)	6.0 (2.0, 9.0)	<0.001
**Personal history**				
Smoking (%)	48 (16.5)	40 (17.7)	8 (12.3)	0.302
Hazardous alcohol consumption (%)	36 (12.4)	33 (14.6)	3 (4.6)	0.031
**Comorbidities**				
Hypertension (%)	77 (26.5)	57 (25.2)	20 (30.8)	0.372
Type 2 diabetes (%)	31 (10.7)	25 (11.1)	6 (9.2)	0.673
Chronic liver diseases (%)	62 (21.3)	57 (25.2)	5 (7.7)	0.002
Chronic lung diseases (%)	9 (3.1)	6 (2.7)	3 (4.6)	0.421
Cardiovascular diseases (%)	9 (3.1)	5 (2.2)	4 (6.2)	0.106
Chronic kidney diseases (%)	3 (1.0)	2 (0.9)	1 (1.5)	0.646
Cerebrovascular disease (%)	24 (8.2)	17 (7.5)	7 (10.8)	0.402
Cancer (%)	10 (3.4)	10 (4.4)	0	0.084
HIV (%)	0	0	0	NA
**Clinical signs and symptoms**				
**Respiratory**				
Dyspnoea (%)	14 (4.8)	4 (1.8)	10 (15.4)	<0.001
Cough (%)	71 (24.4)	55 (24.3)	16 (24.6)	0.963
Sputum (%)	48 (16.5)	38 (16.8)	10 (15.4)	0.784
**Gastrointestinal**				
Diarrhoea (%)	133 (45.7)	101 (44.7)	32 (49.2)	0.517
Abdominal pain (%)	56 (19.2)	44 (19.5)	12 (18.5)	0.856
Vomiting (%)	74 (25.4)	54 (23.9)	20 (30.8)	0.262
Nausea (%)	111 (38.1)	86 (38.1)	25 (38.5)	0.952
Anorexia (%)	256 (88.0)	198 (87.6)	58 (89.2)	0.723
Jaundice (%)	23 (7.9)	16 (7.1)	7 (10.8)	0.331
**Non-specific**				
Fever (%)	289 (99.3)	224 (99.1)	65 (100.0)	0.447
Fatigue (%)	231 (79.4)	178 (78.8)	53 (81.5)	0.626
Muscle ache (%)	94 (32.3)	81 (35.8)	13 (20.0)	0.016
Headache (%)	62 (21.3)	56 (24.8)	6 (9.2)	0.007
Rash (%)	0	0	0	NA
**Laboratory tests at admission**				
WBC (×10^9^/L)	3.2 (2.0, 5.3)	3.2 (2.0, 5.1)	3.8 (2.4, 6.1)	0.166
Neutrophil (×10^9^/L)	1.8 (0.9, 3.6)	1.8 (0.9, 3.3)	2.3 (1.0, 4.6)	0.174
Lymphocyte (×10^9^/L)	0.9 (0.5, 1.3)	0.9 (0.5, 1.3)	0.9 (0.4, 1.4)	0.447
Hb (g/L)	129.0 (117.0, 141.0)	130.5 (118.0, 141.3)	125.0 (106.0, 141.5)	0.156
PLT (×10^9^/L)	49.0 (34.0, 73.0)	52.0 (38.0, 78.0)	35.0 (25.0, 52.0)	<0.001
ALB (g/L)	33.2 (30.1, 35.9)	33.8 (31.2, 36.1)	30.1 (28.2, 34.0)	<0.001
GLB (g/L)	25.3 (22.0, 30.0)	25.3 (22.2, 30.3)	25.2 (20.5, 29.5)	0.353
Cr (μmol/L)	62.0 (49.0, 82.5)	58.5 (48.0, 73.8)	80.0 (59.0, 125.0)	<0.001
PT (s)	11.4 (10.7, 12.4)	11.2 (10.5, 12.1)	12.2 (11.4, 14.1)	<0.001
INR	1.0 (1.0, 1.1)	1.0 (0.9, 1.1)	1.1 (1.0, 1.2)	<0.001
**Treatment**				
Oxygen therapy (%)	109 (37.5)	76 (33.6)	33 (50.8)	0.012
Noninvasive mechanical ventilation (%)	19 (6.5)	5 (2.2)	14 (21.5)	<0.001
Invasive mechanical ventilation (%)	28 (9.6)	9 (4.0)	19 (29.2)	<0.001
Blood purification (%)^a^	15 (5.2)	4 (1.8)	11 (16.9)	<0.001
Use of corticosteroid (%)^b^	84 (28.9)	48 (21.2)	36 (55.4)	<0.001
Intravenous immunoglobulin treatment (%)	116 (39.9)	79 (35.0)	37 (56.9)	0.001
Use of ribavirin (%)	269 (92.4)	207 (91.6)	62 (95.4)	0.308
Antibiotic therapy (%)	212 (72.9)	154 (68.1)	58 (89.2)	0.001
**Complications**				
Disturbance of consciousness (%)	92 (31.6)	48 (21.2)	44 (67.7)	<0.001
Respiratory failure (%)	43 (14.8)	9 (4.0)	34 (52.3)	<0.001
Shock (%)	30 (10.3)	10 (4.4)	20 (30.8)	<0.001

^a^ Blood purification including hemodialysis and plasma exchange

^b^ Corticosteroid mainly including dexamethasone, hydrocortisone and methylprednisolone

Abbreviations: ALB, albumin; Cr, creatinine; GLB, globulin; Hb, hemoglobin; INR, international normalized ratio; PLT, platelet count; PT, prothrombin time; WBC, white blood cell.

### Liver function of patients with SFTS at admission and during hospitalization

Two hundred and eighty-one (96.6%) patients and 287 (98.6%) patients had liver abnormality at admission and during hospitalization, respectively (Table 2). Two hundred and nine (71.8%) patients suffered from liver injury during hospitalization, of whom 175 (60.1%) already had liver injury at admission. Non-survivors had significantly higher AST (253.0 U/L vs. 131.1 U/L, P<0.001) and ALP (86.2 U/L vs. 67.9 U/L, P = 0.006) levels than survivors at admission, while the ALT (73.9 U/L vs. 84.7 U/L, P = 0.447), GGT (54.5 U/L vs. 86.3 U/L, P = 0.077), and TBil (10.0 μmol/L vs. 9.7 μmol/L, P = 0.508) levels were comparable. The difference of liver indicators at the peak level during hospitalization between two groups was consistent with that at admission. When referring to the elevation of liver indicators, the incidences of elevated AST (93.5% at admission and 95.5% during hospitalization), ALT (79.0% at admission and 90.4% during hospitalization) and GGT (59.1% at admission and 77.7% during hospitalization) levels were common. Notably, only the levels of AST, ALP, and the proportion of elevated ALP were higher in non-survivors at baseline and peak levels compared to survivors.

**Table 2:** The features of demography and liver function tests of patients with severe fever with thrombocytopenia syndrome between survivors and non-survivors.

	All patients (n = 291)	Survivors (n = 226)	Non-survivors (n = 65)	P value
**Liver function at admission**				
ALT (U/L)	76.4 (46.8, 139.7)	73.9 (44.0, 140.0)	84.7 (49.9, 139.4)	0.447
AST (U/L)	152.3 (79.0, 346.2)	131.1 (73.8, 309.2)	253.0 (131.4, 601.1)	<0.001
ALP (U/L)	69.8 (54.9, 106.5)	67.9 (54.3, 95.0)	86.2 (58.7, 137.9)	0.006
GGT (U/L)	58.3 (27.0, 146.4)	54.5 (26.1, 142.5)	86.3 (33.8, 154.7)	0.077
TBil (μmol/L)	9.9 (6.9, 17.5)	10.0 (7.2, 16.9)	9.7 (5.9, 19.0)	0.508
Abnormal liver tests ^a^				
Elevated ALT (%)	230 (79.0)	175 (77.4)	55 (84.6)	0.210
Elevated AST (%)	272 (93.5)	210 (92.9)	62 (95.4)	0.478
Elevated ALP (%)	23 (7.9)	10 (4.4)	13 (20.0)	<0.001
Elevated GGT (%)	172 (59.1)	129 (57.1)	43 (66.2)	0.190
Elevated TBil (%)	32 (11.0)	23 (10.2)	9 (13.8)	0.405
Liver abnormality (%) ^b^	281 (96.6)	217 (96.0)	64 (98.5)	0.340
Liver abnormality type ^c^				
Hepatocellular type (%)	254 (87.3)	204 (90.3)	50 (76.9)	<0.001
Cholestatic type (%)	1 (0.3)	0	1 (1.5)	0.065
Mixed type (%)	22 (7.6)	10 (4.4)	12 (18.5)	<0.001
Others (%)	4 (1.4)	3 (1.3)	1 (1.5)	0.915
Liver injury (%) ^d^	175 (60.1)	124 (54.9)	51 (78.5)	0.001
**Peak levels of liver indictors during hospitalization**				
ALT (U/L)	99.9 (60.3, 176.8)	99.7 (58.7, 174.5)	102.6 (71.3, 178.7)	0.357
AST (U/L)	201.1 (95.0, 405.0)	164.7 (83.8, 349.1)	335.9 (174.8, 863.8)	<0.001
ALP (U/L)	92.4 (70.4, 144.2)	84.6 (68.0, 133.2)	125.2 (85.0, 207.2)	<0.001
GGT (U/L)	108.2 (48.0, 241.2)	108.1 (47.2, 238.1)	110.9 (73.0, 257.3)	0.421
TBil (μmol/L)	21.4 (13.1, 35.9)	22.0 (13.6, 36.0)	19.0 (11.1, 34.2)	0.160
Abnormal liver tests ^a^				
Elevated ALT (%)	263 (90.4)	202 (89.4)	61 (93.8)	0.282
Elevated AST (%)	278 (95.5)	214 (94.7)	64 (98.5)	0.195
Elevated ALP (%)	45 (15.5)	24 (10.6)	21 (32.3)	<0.001
Elevated GGT (%)	226 (77.7)	170 (75.2)	56 (86.2)	0.062
Elevated TBil (%)	53 (18.2)	38 (16.8)	15 (23.1)	0.249
Liver abnormality (%) ^b^	287 (98.6)	222 (98.2)	65 (100.0)	0.280
Liver abnormality type ^c^				
Hepatocellular type (%)	232 (80.8)	191 (86.0)	41 (63.1)	<0.001
Cholestatic type (%)	0	0	0	-
Mixed type (%)	46 (16.0)	24 (10.8)	22 (33.8)	<0.001
Others (%)	3 (1.0)	2 (0.9)	1 (1.5)	0.657
Liver injury (%) ^d^	209 (71.8)	152 (67.3)	57 (87.7)	0.001

^a^ An abnormal liver test was defined as an increase of parameters of liver function tests above the upper limit of normal (ULN). The ULNs of each liver function test parameter are: total bilirubin (TBil) = 28 μmol/L, alanine aminotransferase (ALT) = 40 U/L, aspartate aminotransferase (AST) = 40 U/L, alkaline phosphatase (ALP) = 185 U/L, and γ-glutamyl transpeptidase (GGT) = 50 U/L for male and 35 for female.

^b^ Any of the above indicators of liver function tests exceed the ULNs would be considered as liver abnormality.

^c^ Liver abnormalities patterns: hepatocellular type: with ALT or AST > 40 U/L and ALP < 185 U/L; cholestatic type: with ALP > 185 U/L but both ALT and AST < 40 U/L; mixed type refers to ALP > 185 U/L and ALT/AST< 40 U/L; Others refer to patterns not matched with types of liver abnormalities mentioned above.

^d^ Liver injury was defined as an elevation in ALT or AST of at least 3×ULN, or an elevation in ALP or TBil of at least 2×ULN.

Abbreviations: ALP, alkaline phosphatase; ALT, alanine aminotransferase; AST, aspartate aminotransferase; GGT, gama-glutamyl transpeptidase; TBil, total bilirubin.

Two hundred and fifty-four (87.3%) patients at admission and 232 (80.8%) patients during hospitalization had hepatocellular type of liver abnormality. One (0.3%) patient at admission and none during hospitalization had cholestatic type. Twenty-two (7.6%) patients at admission and 46 (16.0%) patients during hospitalization had mixed type. The incidence of hepatocellular type of liver abnormality was much higher in survivors compared to non-survivors (90.3% vs. 76.9%, P<0.001), while the proportion of mixed type was higher in non-survivors (4.4% vs. 18.5%, P<0.001). The prevalence of liver abnormalities between survivors and non-survivors was comparable. However, the incidence of liver injury in non-survivors was significantly higher than that in survivors both at admission (78.5% vs. 54.9%, P = 0.001) and during hospitalization (87.7% vs. 67.3%, P = 0.001).

The last liver function tests of patients during hospitalization were shown in S1 Table. One hundred and ninety (84.1%) and 34 (15.0%) patients had liver abnormalities and liver injury at discharge, respectively. The elevation of ALT (58.0%) and GGT (59.7%) were most common at discharge. However, as high as 96.9% and 67.7% of deceased SFTS patients showed liver abnormalities and liver injury in their last liver function tests, respectively. The elevation of AST (84.6%) and ALT (83.1%) were most commonly observed in deceased SFTS patients before succumbing.

We further compared the mortality rate of specific liver indicators stratified by the elevation degree (S2 Table). Patients with ALP > 2×ULN at admission had the highest mortality rate (66.7%), followed by patients with TBil > 2×ULN (33.3%), and AST > 5×ULN (31.7%). Similarly, patients with peak level of ALP > 2×ULN and AST > 5×ULN had high mortality rate of 70.0% and 30.1%, respectively.

### Dynamic change of liver function indicators after hospital admission in patients with SFTS

**Fig 1:**
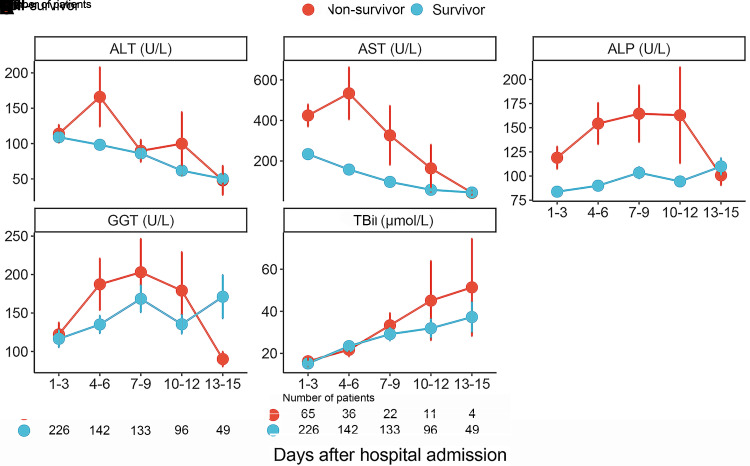
Dynamic changes of liver function indicators after admission between survivors and non-survivors.

The dynamic change of liver function indicators during hospitalization was analyzed to further determine the impact of liver function indicators on prognosis. Fig 1 showed the dynamic changes of liver function indicators in survivors and non-survivors. As mentioned above, the mean levels of ALT, GGT, and TBil between two groups were comparable at admission. However, the ALT and AST levels in non-survivors increased significantly within 4–6 days after admission, while survivors had steady decrease after admission. Notably, non-survivors had an increasing ALP level after admission whereas survivors had a relatively stable level. The GGT reached peak level within 7–9 days after admission and then decreased gradually, while the TBil showed an increasing trend during hospitalization in both groups.

### Association of liver function and mortality risk in patients with SFTS

**Fig 2:**
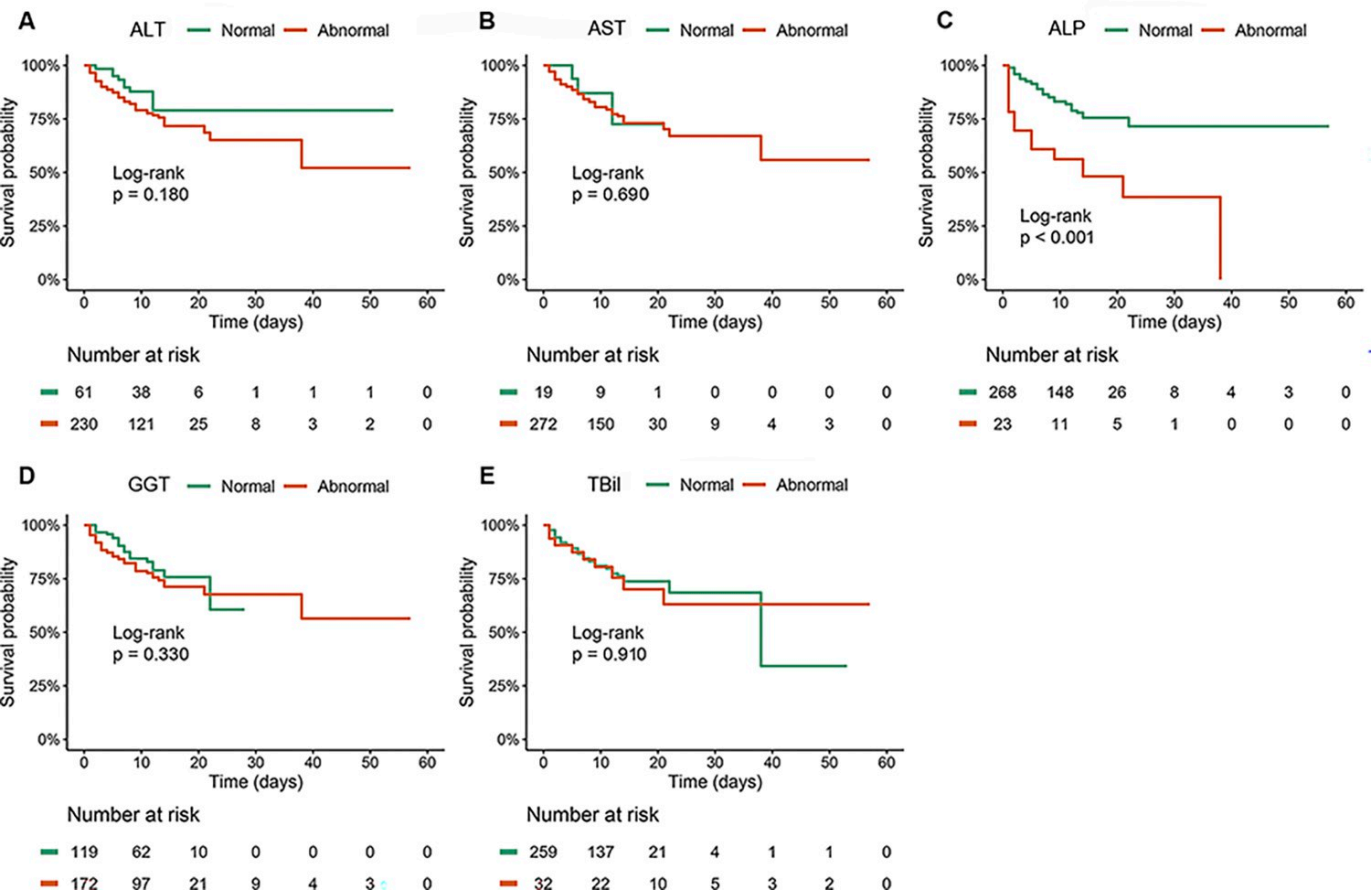
Comparison of cumulative survival rate between patients with and without abnormal liver function indicators.

**Fig 3:**
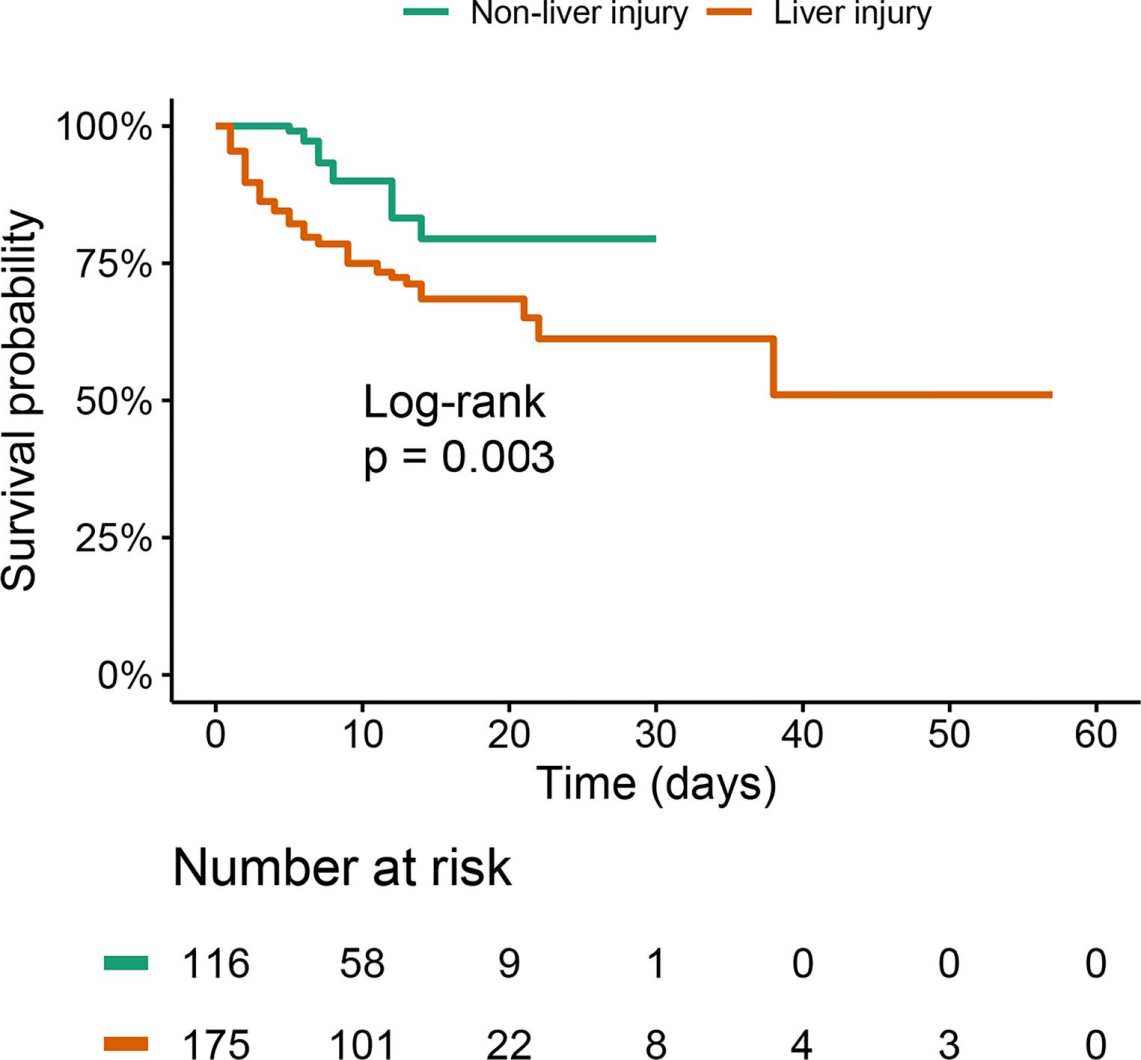
Comparison of cumulative survival rate between patients with and without liver injury at admission.

Cox proportional hazards model was used to explore the association of liver function indicators with in-hospital mortality in SFTS patients (Table 3). In the univariate analysis, older age, WBC, PLT, AST, ALP, Cr, PT, use of corticosteroid and IVIg were associated with mortality. Potential confounders were identified by the directed acyclic graph, which included older age, hazardous alcohol consumption, type 2 diabetes and CLD (S1 Fig). Further multivariate analysis revealed that ALP was the only liver indicator associated with all-cause mortality (HR 1.005, 95%CI 1.002–1.008, P = 0.001), and Cr (HR 1.004, 95%CI 1.000–1.008, P = 0.031), PT (HR 1.085, 95%CI 1.023–1.151, P = 0.007), use of corticosteroid (HR 2.418, 95%CI 1.400–4.175, P = 0.002) were also risk factors of mortality. The Kaplan-Meier analysis (Fig 2C) indicated that patients with elevated ALP at admission had higher cumulative incidence of mortality than those with normal ALP (P<0.001). To determine the association of liver injury at admission with mortality, another Cox proportional hazards model was developed. The presence of liver injury (HR 2.049, 95%CI 1.058–3.966, P = 0.033) was found to be associated with mortality risk after adjusting other confounding factors. Fig 3 also revealed that patients with liver injury at admission had higher cumulative mortality risk than those without liver injury (P = 0.003).

**Table 3:** The cox regression analysis of mortality risk for the patients with severe fever with thrombocytopenia syndrome.

	Univariate		Multivariate-1		Multivariate-2	
	HR (95% CI)	P value	HR (95% CI)	P value	HR (95% CI)	P value
Age (yr)	1.042 (1.019, 1.066)	<0.001	1.059 (1.032, 1.088)	<0.001	1.052 (1.026, 1.079)	<0.001
Sex						
Female	Reference					
Male	0.828 (0.508, 1.350)	0.449				
Hypertension						
No	Reference					
Yes	1.163 (0.682, 1.982)	0.58				
Type 2 diabetes						
No	Reference		Reference		Reference	
Yes	0.835 (0.360, 1.935)	0.674	0.733 (0.310, 1.732)	0.479	0.864 (0.367, 2.035)	0.738
Hazardous alcohol consumption						
No	Reference		Reference		Reference	
Yes	0.334 (0.105, 1.066)	0.064	0.309 (0.094, 1.021)	0.054	0.328 (0.099, 1.088)	0.068
Chronic liver diseases	0.287 (0.115, 0.718)	0.008	0.445 (0.175, 1.132)	0.089	0.446 (0.175, 1.137)	0.446
WBC (×10^9^/L)	1.060 (1.002, 1.121)	0.044	1.028 (0.962, 1.098)	0.419	1.030 (0.966, 1.099)	0.364
PLT (×10^9^/L)	0.987 (0.978, 0.997)	0.012	0.996 (0.987, 1.006)	0.448	0.999 (0.990, 1.008)	0.800
ALT (U/L)	1.000 (0.998, 1.003)	0.754				
AST (U/L)	1.001 (1.000, 1.001)	0.001	1.000 (1.000, 1.001)	0.484		
ALP (U/L)	1.006 (1.003, 1.008)	<0.001	1.005 (1.002, 1.008)	0.001		
GGT (U/L)	1.000 (0.998, 1.001)	0.891				
TBil (μmol/L)	0.999 (0.987, 1.011)	0.853				
Liver injury						
No	Reference				Reference	
Yes	2.391 (1.320, 4.332)	0.004			2.049 (1.058, 3.966)	0.033
Cr (μmol/L)	1.006 (1.003, 1.008)	<0.001	1.004 (1.000, 1.008)	0.031	1.004 (1.000, 1.007)	0.048
PT (s)	1.126 (1.071, 1.184)	<0.001	1.085 (1.023, 1.151)	0.007	1.083 (1.027, 1.142)	0.003
Use of ribavirin						
No	Reference		Reference		Reference	
Yes	1.878 (0.586, 6.021)	0.289	2.485 (0.583, 10.598)	0.219	3.286 (0.770, 14.032)	0.108
Use of corticosteroid						
No	Reference		Reference		Reference	
Yes	3.072 (1.876, 5.031)	<0.001	2.418 (1.400, 4.175)	0.002	2.339 (1.364, 4.012)	0.002
Intravenous immunoglobulin treatment						
No	Reference		Reference		Reference	
Yes	1.863 (1.136, 3.053)	0.014	1.143 (0.670, 1.950)	0.624	1.294 (0.764, 2.192)	0.337

ALP, alkaline phosphatase; ALT, alanine aminotransferase; AST, aspartate aminotransferase; CI, confidence interval; Cr, creatinine; GGT, gama-glutamyl transpeptidase; HR, hazard ratio; PLT, platelet count; PT, prothrombin time; TBil, total bilirubin; WBC, white blood cell.

### The impact of chronic liver diseases on the survival outcome of patients with SFTS

The impact of chronic liver diseases (CLD) on the patients with SFTS was further analyzed. Our cohort included a total of 62 patients with CLD, with 57 patients in the survived group and 5 patients in the deceased group. Specifically, 20 cases were attributed to chronic hepatitis B (CHB), 35 cases to non-alcoholic fatty liver disease (NAFLD), 6 cases to alcoholic fatty liver disease, and 1 case to chronic hepatitis C with elevated liver enzymes. Only two patients had liver cirrhosis and both patients were categorized as Child-Pugh class A. The baseline characteristics of patients with SFTS with and without CLD were compared (S3 Table). SFTS patients without CLD were significantly older than patients with CLD (64.0 years vs. 55.5 years, P = 0.002). S2 Fig showed the dynamic change of liver indicators in patients with and without CLD. The levels of AST and ALT in both groups tended to decrease gradually during hospitalization, while the levels of GGT and TBil were generally increasing. SFTS patients with CLD tended to have an increasing trend of ALP level whereas those without CLD had a stable level. Among SFTS patients without CLD, the dynamic change of liver indicators had a similar trend with the whole cohort, whereas the changing features of liver indicators in those with CLD were quite different from general patients (S3 Fig).

The Kaplan-Meier analysis was adopted to compare the survival difference among patients with and without CLD (S4A Fig). SFTS patients with CLD had lower cumulative incidence of mortality than those without CLD (P = 0.004). However, the presence of CLD was not an independent risk factor of fatal outcome after adjusting for the confounders (Table 3). We further investigated the survival difference in subgroups stratified by the presence of liver injury. Among SFTS patients without CLD, the cumulative mortality of patients with liver injury showed higher cumulative incidence of mortality than those without liver injury (P = 0.003). However, the cumulative mortality risk was comparable between patients with and without liver injury in patients with CLD. The mortality risk for SFTS patients without CLD was also analyzed using Cox regression analysis (S4 Table). Abnormal ALP (HR 1.006, 95%CI 1.003–1.009, P<0.001) and the presence of liver injury (HR 2.152, 95%CI 1.077–4.300, P = 0.030) were associated with higher mortality in multivariate analysis. The potential role of CLD as a moderator was investigated using a two-factor ANOVA, yielding a P-value for interaction term of 0.272.

## Discussion

In this multicenter study, we analyzed the association of liver injury with clinical prognosis in SFTS patients. As high as 96.6% and 60.1% of SFTS patients had liver abnormalities and liver injury on admission, respectively. The prevalence of liver injury was significantly higher in the deceased group. Moreover, the incidence of liver injury, especially ALP elevation, was a risk factor of fatal outcome in SFTS patients.

The mortality rate of patients with SFTS was 22.3% in this study, which was similar to previous studies [3,7]. SFTS patients with older age had a significantly higher mortality risk [22]. Besides age, there were significant differences in PT, Cr, use of corticosteroid and ALP or the presence of liver injury between survivors and non-survivors. SFTS is characterized by hemorrhagic symptoms, with DIC as a major cause of death [10,23]. Previous studies have suggested SFTSV-induced liver injury is associated with the decrease of coagulation factor synthesis [24]. PT is a sensitive and widely used indicator of extrinsic coagulation system. Prolonged PT in SFTS patients indicates reduction of coagulation factors, reflecting impairment of hepatic synthetic function [25]. We also found that PT prolongation is common in the deceased group, with a significantly longer PT than in survivors. The primary reasons for an elevated Cr level may include a reduction in glomerular filtration rate (GFR), decreased blood volume (hypovolemia), and congestive heart failure. Elevated Cr levels may suggest compromised early renal function or inadequate circulating blood volume, which further demonstrates that SFTS is a disease involving multiple systems including kidney and cardiac muscle. The supportive treatment is mainly adopted for SFTS patients in clinical practice, including transfusion of blood products and intensive care for critically ill patients [8]. A greater proportion of patients in the deceased group had received the treatment of mechanical ventilation, blood purification and the use of corticosteroid or IVIg. Antiviral therapy with ribavirin is clinically recommended [26,27] and a previous study [7] found that patients who received ribavirin therapy at early stage had increased survival. However, the proportion of patients with the use of ribavirin was comparable between survivors and non-survivors (91.6% vs. 95.4%, P = 0.308), which was consistent with several previous studies [28,29] showing that ribavirin treatment did not improve the survival rates of patients with SFTS. Therefore, the effectiveness of ribavirin therapy remains inconclusive and further prospective, large-scale studies are required to validate the efficacy of ribavirin in clinical practice. Notably, the use of corticosteroid was positively associated with mortality. However, the use of steroids was common among critically ill patients, which might also reflect the progression of disease. Therefore, it could reflect confounding by indication but may also have implications for the management of SFTS patients.

In our cohort, as high as 60.1% of patients already had liver injury at admission and the proportion increased to 71.8% during hospitalization. Patients with liver injury at admission had higher cumulative incidence of mortality than those without. Our study demonstrated that liver injury was associated with substantially increased mortality risk. The main pattern of liver injury was hepatocellular in both survivors and non-survivors either at admission or during hospitalization. However, the proportion of hepatocellular type in survivors was significantly higher compared to that in non-survivors (90.3% vs. 76.9%, P<0.001). ALT and AST are main indicators to reflect hepatocyte damage, which were also most common liver indicators elevated at baseline and peak level. Of which, only AST showed disparity between survivors and non-survivors, with the degree and proportion of elevation much greater than that of ALT. According to several studies [25,30,31], elevated AST might be an indicator of poor outcome in SFTS. However, the findings of our study are consistent with those of earlier investigations [14,25] in which AST elevation at admission was not a risk factor for mortality. This may be because abnormal liver function is prevalent in the early stage of disease, while more severe liver function damage is found in the middle to late stages of the deceased patients. Compared to ALT, AST had more sources of generation from various tissues [13,32,33] including liver, cardiac muscle, skeletal muscle and brain. Significantly elevated AST, along with increased Cr, may suggest the presence of multiorgan failure [13], which was also the leading cause of death in SFTS patients. Previous studies [34] also showed that the presence of multiorgan failure is more common in the deceased patients with SFTS. Therefore, elevated AST at admission may indicate systemic organ damage rather than severe liver dysfunction, thus not accurately reflecting the association between liver injury and mortality.

The proportion of mixed liver abnormalities in the deceased group was much higher than that in the survival group. ALP [33,35] was considered as the primary enzyme to assess the cholestatic injury. Although patients with elevated ALP account for a relatively small proportion in our study, there existed significant difference between survivors and non-survivors. Our results were consistent with previous studies which showed that elevated ALP was a risk factor for mortality in patients with SFTS [12,14]. Notably, only one patient had cholestatic injury at admission and had a fatal outcome.

The mechanisms of liver injury in SFTS patients are not fully elucidated. However, several studies [36,37] have demonstrated that the liver was one of the primary target organs of SFTS infection due to the presence of a large amount of macrophages which are targeted by SFTSV. Further study [10] demonstrated that human liver epithelial cells were also susceptible to SFTSV, which could directly infect and replicate in liver both in human and mice. Moreover, liver injury may be a secondary immunopathological response in SFTS as proinflammatory cytokines and chemokines were strongly induced upon SFTSV infection, providing an immunopathological basis for liver pathology [10]. However, more studies are needed to explore the mechanism of liver injury and the association with fatal outcome in SFTS.

We further investigated the association of CLD with adverse prognosis in SFTS patients. We found that patients with CLD had higher ALP and TBil level compared to patients without CLD. Surprisingly, we found that SFTS patients with CLD had lower mortality rate. However, patients without CLD were significantly older than patients with CLD. As previous studies [38–40] reported that old age was an important risk of fatal outcome in SFTS. After adjusting for confounders including age, concurrent with CLD was not a risk factor of fatal outcome in SFTS patients. A previous study [14] also demonstrated that concomitant hepatitis B virus infection was not associated with increased risk of mortality in SFTS patients. Among patients without CLD, the presence of liver injury had increased cumulative incidence of mortality. ALP and the presence of liver injury were associated with mortality among SFTS patients without CLD. The mortality in patients with liver injury was comparable with those without liver injury in SFTS patients with CLD. Besides, the presence of CLD did not have a significant moderating effect on the association of liver injury and mortality. However, given the small number of patients with CLD in our cohort, more studies are needed to investigate the association of liver injury in SFTS patients with CLD.

There are also several limitations in this study. First, only hospitalized SFTS patients were included in the present study and patients treated in outpatients were not included. Thus, the present study might bias towards more severe patients. The findings need to be validated in SFTS patients treated in outpatients. Second, potential causes of abnormal liver function, including the use of hepatotoxic drugs and the presence of biliary tract diseases, might bias our results. Third, analyzing alterations in liver function markers before and after infection could offer insights into the impact of SFTS on liver function and disease progression. However, we did not have liver function test results for these patients before SFTS virus infection. Fourth, cirrhosis represents a distinct pathophysiological process from acute liver injury and should be regarded as a significant risk factor. Due to the limited sample size of patients with cirrhosis, we were unable to analyze the association between cirrhosis and survival outcomes. Fifth, due to the lack of sufficient data, we could not calculate the Charlson Comorbidity Index and Glasgow Coma Scale scores to fully evaluate the patients’ underlying condition. In addition, this study is retrospective and the sample size is relatively small. Therefore, further multicenter, prospective studies are required to better assess the impact of liver injury on prognosis.

In conclusion, patients with SFTS are often associated with pronounced liver injury and the presence of liver injury is strongly associated with poor prognosis in SFTS patients. Elevated ALP was an independent predictor for poor prognosis. Therefore, liver function should be closely monitored in SFTS patients during hospitalization. However, due to the lack of specific antiviral drugs for SFTS, supportive care is especially important for these patients. The monitor of liver function and evaluation of liver injury may help the physicians to identify patients who had higher mortality risk and provide better supportive care to improve clinical outcomes. Further studies are imperative to explore the underlying mechanisms and optimize treatment strategies to improve the outcomes for SFTS patients with liver injury.

## Supporting information

S1 Table Last liver function of patients with severe fever with thrombocytopenia syndrome between survivors and non-survivors during hospitalization. (DOCX)

S2 Table Mortality rate of patients with severe fever with thrombocytopenia syndrome according to abnormality of liver function tests. (DOCX)

S3 Table The features of demography and liver function of patients with severe fever with thrombocytopenia syndrome between patients with and without chronic liver diseases. (DOCX)

S4 Table The Cox regression analysis of mortality risk for the patients with severe fever with thrombocytopenia syndrome without chronic liver diseases. (DOCX)

S1 Fig The directed acyclic graph derived from prior knowledge and descriptive statistics. (TIF)

S2 Fig Dynamic changes of liver indicators after admission between patients with and without chronic liver diseases. (TIF)

S3 Fig Dynamic changes of liver indicators after admission between survivors and non-survivors with or without chronic liver diseases. (TIF)

S4 Fig Comparison of cumulative survival rate between patients with and without chronic liver diseases (A), patients with and without liver injury in chronic liver diseases group (B) and without chronic liver diseases group (C).
(TIF)
