# Supplementary material for: Association of liver function and prognosis in patients with severe fever with thrombocytopenia syndrome
Source: PLoS Negl Trop Dis. 2024 Apr 16;18(4):e0012068. doi: 10.1371/journal.pntd.0012068 (PMC11051684; doi:10.1371/journal.pntd.0012068)
Supplement: S1 Table — (DOCX) [file pntd.0012068.s001.docx]

**Last liver function of patients with severe fever with thrombocytopenia syndrome between survivors and non-survivors during hospitalization.**

|  | **All patients (n=291)** | **Survivors (n=226)** | **Non-survivors (n=65)** | **P value** |
| --- | --- | --- | --- | --- |
| ALT (U/L) | 52.0 (30.3, 83.4) | 46.8 (26.9, 71.1) | 80.3 (51.5, 125.8) | <0.001 |
| AST (U/L) | 36.8 (24.3, 91.5) | 31.6 (22.1, 57.2) | 253.0 (60.8, 636.1) | <0.001 |
| ALP (U/L) | 81.9 (64.7, 116.2) | 77.0 (63.4, 102.6) | 122.0 (82.4, 190.0) | <0.001 |
| GGT (U/L) | 80.5 (40.0, 164.1) | 73.1 (38.4, 148.0) | 98.5 (67.8, 198.2) | 0.015 |
| TBil (μmol/L) | 16.0 (10.9, 24.0) | 15.3 (10.9, 23.9) | 17.2 (10.5, 28.0) | 0.575 |
| Elevated ALT (%) | 185 (63.6) | 131 (58.0) | 54 (83.1) | <0.001 |
| Elevated AST (%) | 141 (48.5) | 86 (38.1) | 55 (84.6) | <0.001 |
| Elevated ALP (%) | 26 (8.9) | 9 (4.0) | 17 (26.2) | <0.001 |
| Elevated GGT (%) | 186 (63.9) | 135 (59.7) | 51 (78.5) | 0.006 |
| Elevated TBil (%) | 52 (17.9) | 36 (15.9) | 16 (24.6) | 0.107 |
| Liver abnormality (%) | 253 (86.9) | 190 (84.1) | 63 (96.9) | 0.007 |
| Liver abnormality type |  |  |  |  |
| Hepatocellular type (%) | 174 (68.8) | 131 (68.9) | 43 (68.3) | 0.918 |
| Cholestatic type (%) | 0 | 0 | 0 | - |
| Mixed type (%) | 26 (10.3) | 9 (4.7) | 17 (27.0) | <0.001 |
| Others (%) | 53 (20.9) | 50 (26.3) | 3 (4.8) | <0.001 |
| Liver injury (%) | 78 (26.8) | 34 (15.0) | 44 (67.7%) | <0.001 |

ALP, alkaline phosphatase; ALT, alanine aminotransferase; AST, aspartate aminotransferase; GGT, gama-glutamyl transpeptidase; TBil, total bilirubin.
