# Supplementary material for: Association of liver function and prognosis in patients with severe fever with thrombocytopenia syndrome
Source: PLoS Negl Trop Dis. 2024 Apr 16;18(4):e0012068. doi: 10.1371/journal.pntd.0012068 (PMC11051684; doi:10.1371/journal.pntd.0012068)
Supplement: S2 Table — (DOCX) [file pntd.0012068.s002.docx]

**Mortality rate of patients with severe fever with thrombocytopenia syndrome according to abnormality of liver function tests.**

|  | **Number** | **Death** | **Mortality (%)** | **P value** |
| --- | --- | --- | --- | --- |
| At admission |  |  |  |  |
| ALT |  |  |  | 0.426 |
| ≤ ULN | 61 | 10 | 16.4 |  |
| > 1×ULN to 5×ULN | 193 | 47 | 24.4 |  |
| > 5×ULN | 37 | 8 | 21.6 |  |
| AST |  |  |  | 0.005 |
| ≤ ULN | 19 | 3 | 15.8 |  |
| > 1×ULN to 5×ULN | 149 | 23 | 15.4 |  |
| > 5×ULN | 123 | 39 | 31.7 |  |
| GGT |  |  |  | 0.09 |
| ≤ ULN | 119 | 22 | 18.5 |  |
| > 1×ULN to 5×ULN | 113 | 33 | 28.7 |  |
| > 5×ULN | 57 | 10 | 17.5 |  |
| ALP |  |  |  | <0.001 |
| ≤ ULN | 268 | 52 | 19.4 |  |
| > 1×ULN to 2×ULN | 20 | 11 | 55 |  |
| > 2×ULN | 3 | 2 | 66.7 |  |
| TBil |  |  |  | 0.641 |
| ≤ ULN | 259 | 56 | 21.6 |  |
| > 1×ULN to 2×ULN | 23 | 6 | 26.1 |  |
| > 2×ULN | 9 | 3 | 33.3 |  |
| Peak levels during hospitalization |  |  |  |  |
| ALT |  |  |  | 0.524 |
| ≤ ULN | 28 | 4 | 14.3 |  |
| > 1×ULN to 5×ULN | 207 | 47 | 22.7 |  |
| > 5×ULN | 56 | 14 | 25 |  |
| AST |  |  |  | 0.005 |
| ≤ ULN | 13 | 1 | 7.7 |  |
| > 1×ULN to 5×ULN | 132 | 20 | 15.2 |  |
| > 5×ULN | 146 | 44 | 30.1 |  |
| GGT |  |  |  | 0.114 |
| ≤ ULN | 65 | 9 | 13.8 |  |
| > 1×ULN to 5×ULN | 142 | 38 | 26.8 |  |
| > 5×ULN | 84 | 18 | 21.4 |  |
| ALP |  |  |  | <0.001 |
| ≤ ULN | 246 | 44 | 17.9 |  |
| > 1×ULN to 2×ULN | 35 | 14 | 40 |  |
| > 2×ULN | 10 | 7 | 70 |  |
| TBil |  |  |  | 0.17 |
| ≤ ULN | 192 | 47 | 24.5 |  |
| > 1×ULN to 2×ULN | 65 | 9 | 13.8 |  |
| > 2×ULN | 34 | 9 | 26.5 |  |

ALP, alkaline phosphatase; ALT, alanine aminotransferase; AST, aspartate aminotransferase; GGT, gama-glutamyl transpeptidase; TBil, total bilirubin; ULN, upper limit of normal value.
