# Supplementary material for: Association of liver function and prognosis in patients with severe fever with thrombocytopenia syndrome
Source: PLoS Negl Trop Dis. 2024 Apr 16;18(4):e0012068. doi: 10.1371/journal.pntd.0012068 (PMC11051684; doi:10.1371/journal.pntd.0012068)
Supplement: S3 Table — (DOCX) [file pntd.0012068.s003.docx]

**The features of demography and liver function of patients with severe fever with thrombocytopenia syndrome between patients with and without chronic liver diseases.**

|  | **All patients (n=291)** | **With CLD (n=62)** | **Without CLD (n=229)** | **P value** |
| --- | --- | --- | --- | --- |
| Age (yr) | 63.0 (52.0, 71.0) | 55.5 (48.0, 66.3) | 64.0 (52.5, 72.0) | 0.002 |
| Male (%) | 151 (51.9) | 33 (53.2) | 118 (51.5) | 0.812 |
| **Liver function at admission** |  |  |  |  |
| ALT (U/L) | 76.4 (46.8, 139.7) | 92.8 (58.1, 147.6) | 74.4 (42.0, 139.6) | 0.112 |
| AST (U/L) | 152.3 (79.0, 346.2) | 166.1 (91.0, 343.9) | 150.2 (77.3, 347.0) | 0.745 |
| ALP (U/L) | 69.8 (54.9, 106.5) | 78.2 (55.3, 117.6) | 69.0 (54.7, 97.5) | 0.279 |
| GGT (U/L) | 58.3 (27.0, 146.4) | 78.7 (40.5, 218.2) | 52.2 (26.6, 133.1) | 0.009 |
| TBil (μmol/L) | 9.9 (6.9, 17.5) | 12.4 (7.9, 21.8) | 9.6 (6.8, 16.4) | 0.017 |
| Abnormal liver tests ^a^ |  |  |  |  |
| Elevated ALT (%) | 230 (79.0) | 55 (88.7) | 175 (76.4) | 0.035 |
| Elevated AST (%) | 272 (93.5) | 59 (95.2) | 213 (93.0) | 0.751 |
| Elevated ALP (%) | 23 (7.9) | 4 (6.5) | 19 (8.3) | 0.832 |
| Elevated GGT (%) | 172 (59.1) | 46 (74.2) | 126 (55.0) | 0.006 |
| Elevated TBil (%) | 32 (11.0) | 8 (12.9) | 24 (10.5) | 0.589 |
| Liver abnormality (%) ^b^ | 281 (96.6) | 59 (95.2) | 222 (96.9) | 0.772 |
| Liver abnormality type ^c^ |  |  |  |  |
| Hepatocellular type (%) | 254 (87.3) | 55 (88.7) | 199 (86.9) | 0.704 |
| Cholestatic type (%) | 1 (0.3) | 0 | 1 (0.4) | 1.000 |
| Mixed type (%) | 22 (7.6) | 4 (6.5) | 18 (6.6) | 0.919 |
| Others (%) | 4 (1.4) | 0 | 4 (1.7) | 0.665 |
| Liver injury (%) ^d^ | 175 (60.1) | 37 (59.7) | 183 (79.9) | 0.934 |
| **Peak levels of liver indictors during hospitalization** |  |  |  |  |
| ALT (U/L) | 99.9 (60.3, 176.8) | 101.2 (62.7, 159.2) | 99.8 (56.2, 176.9) | 0.766 |
| AST (U/L) | 201.1 (95.0, 405.0) | 196.9 (93.3, 373.1) | 201.3 (95.5, 419.5) | 0.554 |
| ALP (U/L) | 92.4 (70.4, 144.2) | 103.9 (75.8, 155.4) | 88.0 (69.2, 143.2) | 0.288 |
| GGT (U/L) | 108.2 (48.0, 241.2) | 168.2 (59.6, 329.0) | 99.3 (47.3, 220.9) | 0.029 |
| TBil (μmol/L) | 21.4 (13.1, 35.9) | 22.7 (14.2, 36.8) | 21.0 (12.9, 35.3) | 0.486 |
| Abnormal liver tests ^a^ |  |  |  |  |
| Elevated ALT (%) | 263 (90.4) | 57 (91.9) | 206 (90.0) | 0.639 |
| Elevated AST (%) | 278 (95.5) | 59 (95.2) | 219 (95.6) | 0.874 |
| Elevated ALP (%) | 45 (15.5) | 11 (17.7) | 34 (14.8) | 0.576 |
| Elevated GGT (%) | 226 (77.7) | 51 (82.3) | 175 (76.4) | 0.327 |
| Elevated TBil (%) | 53 (18.2) | 10 (16.1) | 43 (18.8) | 0.632 |
| Liver abnormality (%) ^b^ | 287 (98.6) | 60 (96.8) | 227 (99.1) | 0.200 |
| Liver abnormality type ^c^ |  |  |  |  |
| Hepatocellular type (%) | 232 (80.8) | 48 (86.0) | 182 (63.1) | 0.724 |
| Cholestatic type (%) | 0 | 0 | 0 | - |
| Mixed type (%) | 46 (16.0) | 11 (10.8) | 35 (33.8) | 0.638 |
| Others (%) | 3 (1.0) | 0 (0.9) | 3 (1.5) | 0.413 |
| Liver injury (%) ^d^ | 209 (71.8) | 43 (67.3) | 166 (87.7) | 0.627 |

^a^ An abnormal liver test was defined as an increase of parameters of liver function tests above the upper limit of normal (ULN). The ULNs of each liver function test parameter are: total bilirubin (TBil)=28 µmol/L, alanine aminotransferase (ALT)=40 U/L, aspartate aminotransferase (AST)=40 U/L, alkaline phosphatase (ALP)=185 U/L, and γ-glutamyl transpeptidase (GGT)=50 U/L for male and 35 for female.

^b^ Any of the above indicators of liver function tests exceed the ULNs would be considered as liver abnormality.

^c^ Liver abnormalities patterns: hepatocellular type: with ALT or AST > 40 U/L and ALP < 185 U/L; cholestatic type: with ALP > 185 U/L but both ALT and AST < 40 U/L; mixed type refers to ALP > 185 U/L and ALT/AST< 40 U/L; Others refer to patterns not matched with types of liver abnormalities mentioned above.

^d^ Liver injury was defined as an elevation in ALT or AST of at least 3×ULN, or an elevation in ALP or TBil of at least 2×ULN.

Abbreviations: ALP, alkaline phosphatase; ALT, alanine aminotransferase; AST, aspartate aminotransferase; GGT, gama-glutamyl transpeptidase; TBil, total bilirubin.
