# Supplementary material for: Association of liver function and prognosis in patients with severe fever with thrombocytopenia syndrome
Source: PLoS Negl Trop Dis. 2024 Apr 16;18(4):e0012068. doi: 10.1371/journal.pntd.0012068 (PMC11051684; doi:10.1371/journal.pntd.0012068)
Supplement: S4 Table — (DOCX) [file pntd.0012068.s004.docx]

**The Cox regression analysis of mortality risk for the patients with severe fever with thrombocytopenia syndrome without chronic liver diseases.**

|  | **Univariate** | | **Multivariate** | | **Multivariate** | |
| --- | --- | --- | --- | --- | --- | --- |
|  | **HR (95% CI)** | **P value** | **HR (95% CI)** | **P value** | **HR (95% CI)** | **P value** |
| Age (yr) | 1.042 (1.018, 1.066) | 0.001 | 1.066 (1.037, 1.096) | <0.001 | 1.060 (1.032, 1.088) | <0.001 |
| Sex |  |  |  |  |  |  |
| Female | Reference |  |  |  |  |  |
| Male | 0.896 (0.539, 1.491) | 0.673 |  |  |  |  |
| Hypertension |  |  |  |  |  |  |
| No | Reference |  |  |  |  |  |
| Yes | 1.233 (0.701, 2.168) | 0.467 |  |  |  |  |
| Type 2 diabetes |  |  |  |  |  |  |
| No | Reference |  | Reference |  | Reference |  |
| Yes | 0.743 (0.296, 1.865) | 0.527 | 0.649 (0.255, 1.654) | 0.365 | 0.773 (0.304, 1.967) | 0.589 |
| Hazardous alcohol consumption |  |  |  |  |  |  |
| No | Reference |  | Reference |  | Reference |  |
| Yes | 0.390 (0.122, 1.250) | 0.113 | 0.385 (0.116, 1.274) | 0.118 | 0.460 (0.138, 1.529) | 0.205 |
| WBC (×10^9^/L) | 1.053 (0.995, 1.116) | 0.075 |  |  |  |  |
| PLT (×10^9^/L) | 0.989 (0.980, 0.999) | 0.028 | 0.997 (0.989, 1.006) | 0.525 | 1.000 (0.991, 1.008) | 0.921 |
| ALT (U/L) | 1.000 (0.998, 1.003) | 0.747 |  |  |  |  |
| AST (U/L) | 1.000 (1.000, 1.001) | 0.017 | 1.000 (1.000, 1.001) | 0.716 |  |  |
| ALP (U/L) | 1.005 (1.003, 1.008) | <0.001 | 1.006 (1.003, 1.009) | <0.001 |  |  |
| GGT (U/L) | 1.000 (0.999, 1.002) | 0.822 |  |  |  |  |
| TBil (μmol/L) | 1.000 (0.989, 1.011) | 0.989 |  |  |  |  |
| Liver injury |  |  |  |  |  |  |
| No | Reference |  |  |  | Reference |  |
| Yes | 2.418 (1.303, 4.487) | 0.005 |  |  | 2.152 (1.077, 4.300) | 0.030 |
| Cr (μmol/L) | 1.005 (1.003, 1.008) | <0.001 | 1.004 (1.001, 1.008) | 0.014 | 1.004 (1.001, 1.008) | 0.024 |
| PT (s) | 1.107 (1.050, 1.168) | <0.001 | 1.083 (1.020, 1.150) | 0.009 | 1.079 (1.023, 1.139) | 0.005 |
| Use of ribavirin |  |  |  |  |  |  |
| No | Reference |  | Reference |  | Reference |  |
| Yes | 1.664 (0.521, 5.318) | 0.39 | 2.161 (0.492, 9.504) | 0.308 | 2.988 (0.681, 13.114) | 0.147 |
| Use of corticosteroid |  |  |  |  |  |  |
| No | Reference |  | Reference |  | Reference |  |
| Yes | 2.694 (1.611, 4.505) | <0.001 | 2.300 (1.307, 4.048) | 0.004 | 2.270 (1.300, 3.964) | 0.004 |
| Intravenous immunoglobulin treatment |  |  |  |  |  |  |
| No | Reference |  |  |  |  |  |
| Yes | 1.632 (0.975, 2.729) | 0.062 |  |  |  |  |

ALP, alkaline phosphatase; ALT, alanine aminotransferase; AST, aspartate aminotransferase; CI, confidence interval; Cr, creatinine; GGT, gama-glutamyl transpeptidase; HR, hazard ratio; PLT, platelet count; PT, prothrombin time; TBil, total bilirubin; WBC, white blood cell.
