# Supplementary figures and images for: Association of liver function and prognosis in patients with severe fever with thrombocytopenia syndrome
Source: PLoS Negl Trop Dis. 2024 Apr 16;18(4):e0012068. doi: 10.1371/journal.pntd.0012068 (PMC11051684; doi:10.1371/journal.pntd.0012068)

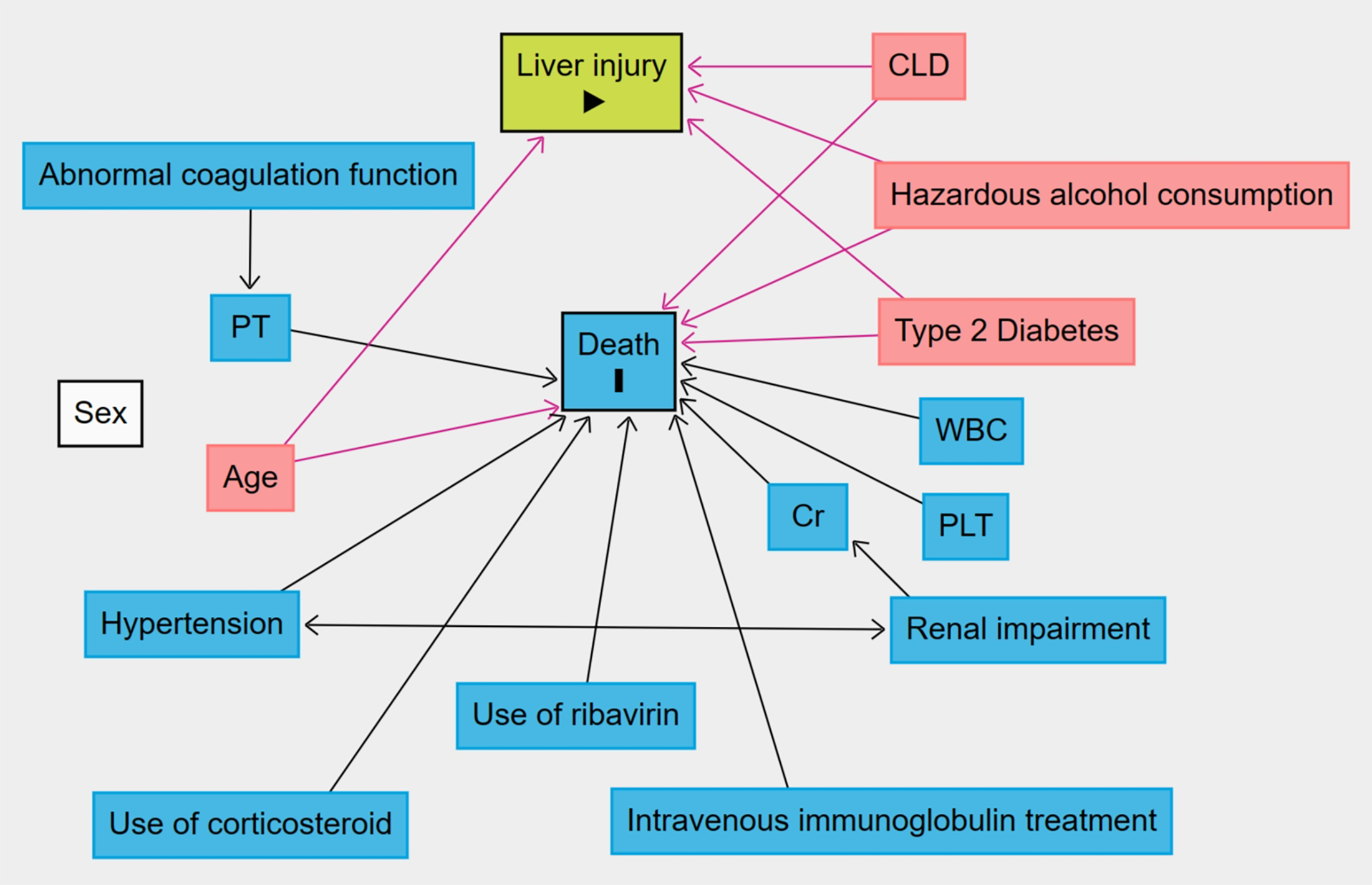

Supplement: S1 Fig — (TIF) [file pntd.0012068.s005.tif]

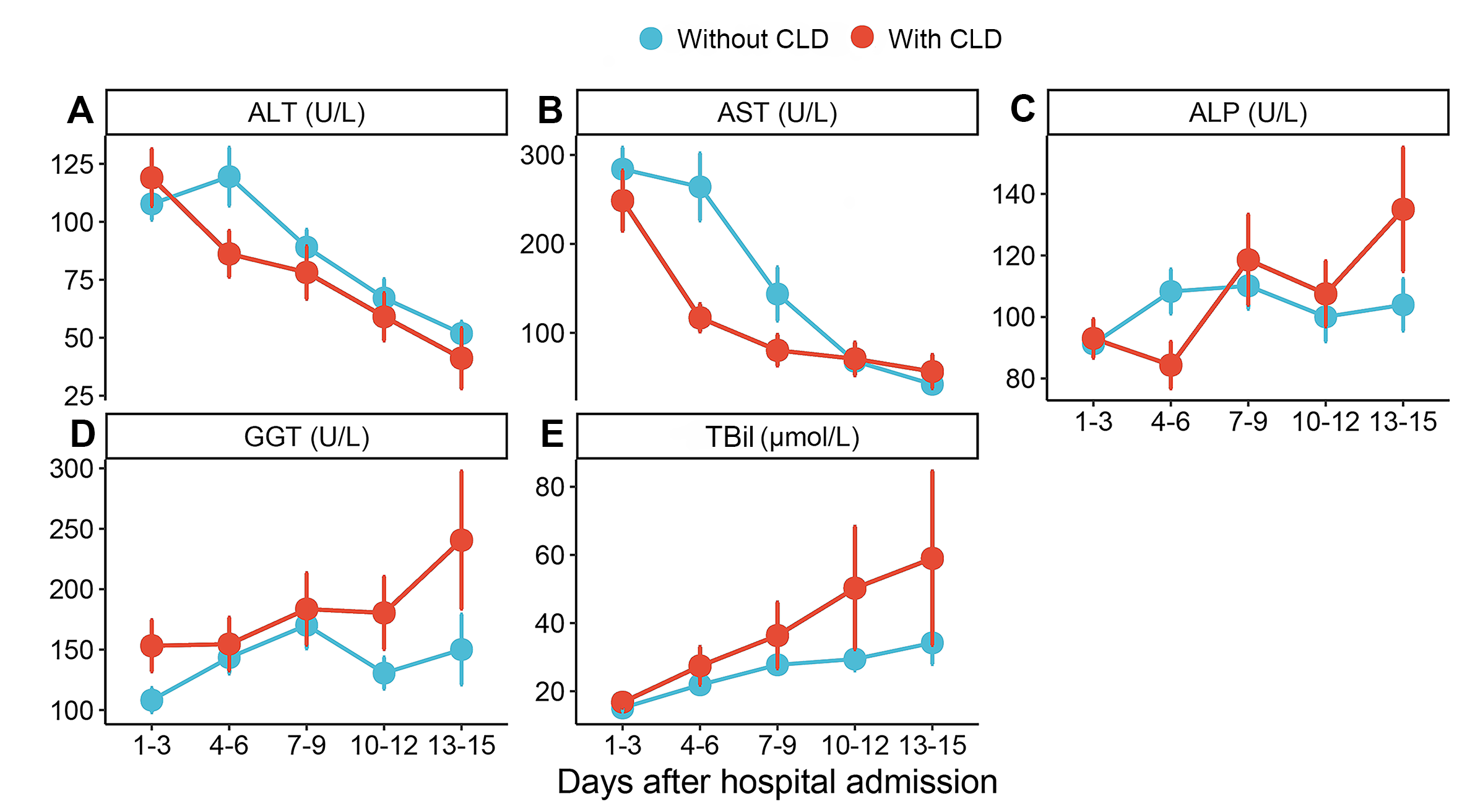

Supplement: S2 Fig — (TIF) [file pntd.0012068.s006.tif]

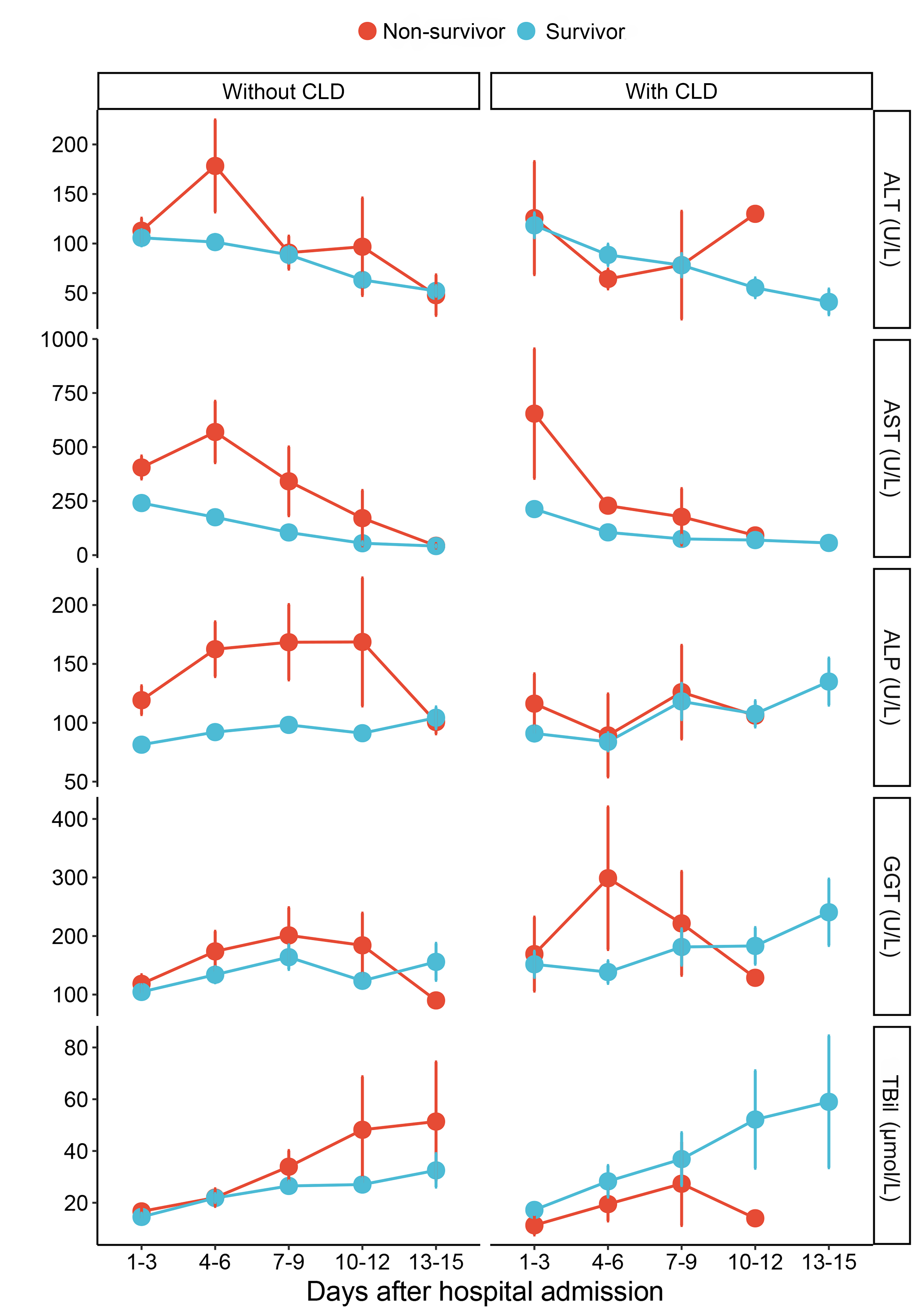

Supplement: S3 Fig — (TIF) [file pntd.0012068.s007.tif]

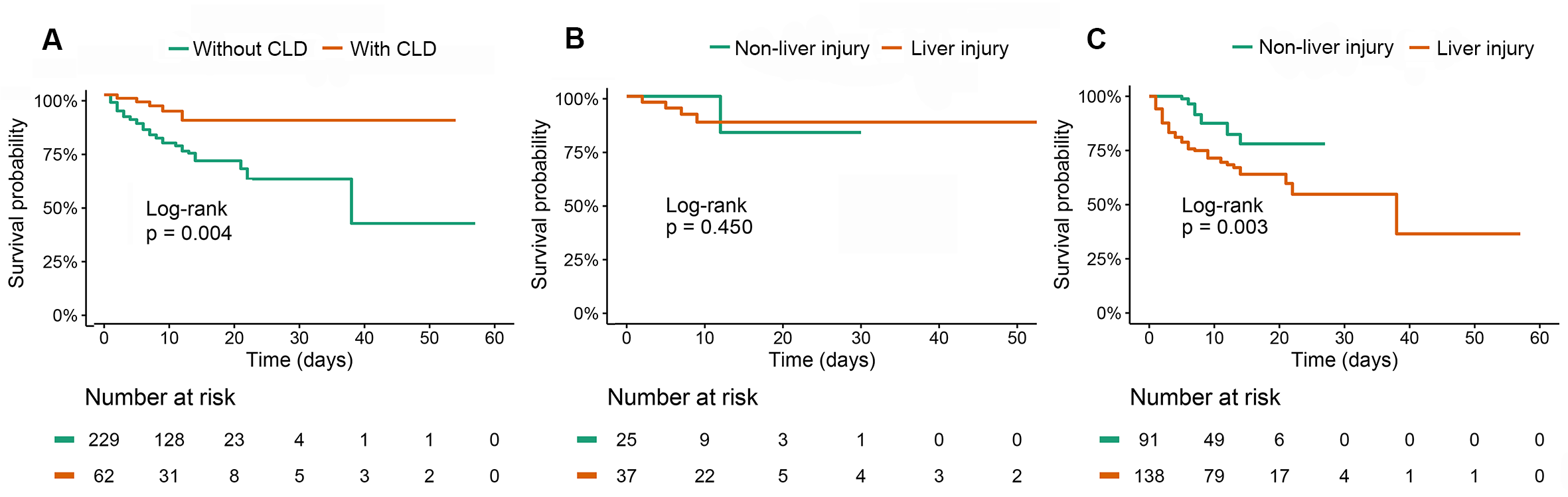

Supplement: S4 Fig — Comparison of cumulative survival rate between patients with and without chronic liver diseases (A), patients with and without liver injury in chronic liver diseases group (B) and without chronic liver diseases group (C). (TIF) [file pntd.0012068.s008.tif]
